# Evaluation of a Web-Based Dietary Assessment Tool (myfood24) in Norwegian Women and Men Aged 60-74 Years: Usability Study

**DOI:** 10.2196/35092

**Published:** 2022-03-11

**Authors:** Laila Arnesdatter Hopstock, Anine Christine Medin, Guri Skeie, André Henriksen, Marie Wasmuth Lundblad

**Affiliations:** 1 Department of Community Medicine UiT The Arctic University of Norway Tromsø Norway; 2 Department of Nutrition and Public Health University of Agder Kristiansand Norway; 3 Department of Computer Science UiT The Arctic University of Norway Tromsø Norway

**Keywords:** system usability score, older adults, measurements, nutrition, dietary intake, digital health, web tool

## Abstract

**Background:**

A healthy diet throughout the life course improves health and reduces the risk of disease. There is a need for new knowledge of the relation between diet and health, but existing methods to collect information on food and nutrient intake have their limitations. Evaluations of new tools to assess dietary intake are needed, especially in old people, where the introduction of new technology might impose challenges.

**Objective:**

We aimed to examine the usability of a new web-based dietary assessment tool in older adult women and men.

**Methods:**

A total of 60 women and men (participation 83%, 57% women) aged 60-74 years recruited by convenience and snowball sampling completed a 24-hour web-based dietary recall using the newly developed Norwegian version of Measure Your Food On One Day (myfood24). Total energy and nutrient intakes were calculated in myfood24, primarily on the basis of the Norwegian Food Composition Table. No guidance or support was provided to complete the recall. Usability was assessed using the system usability scale (SUS), where an SUS score of ≥68 was considered satisfactory. We examined the responses to single SUS items and the mean (SD) SUS score in groups stratified by sex, age, educational level, and device used to complete the recall (smartphone, tablet device, or computer).

**Results:**

The mean total energy intake was 5815 (SD 3093) kJ. A total of 14% of participants had an energy intake of <2100 kJ (ie, 500 kilocalories) and none had an intake of >16,800 kJ (ie, 4000 kilocalories). Mean energy proportions from carbohydrates, fat, protein, alcohol, and fiber was within the national recommendations. The mean SUS score was 55.5 (SD 18.6), and 27% of participants had SUS scores above the satisfactory product cut-off. Higher SUS scores were associated with younger age and lower education, but not with the type of device used.

**Conclusions:**

We found the overall usability of a new web-based dietary assessment tool to be less than satisfactory in accordance with standard usability criteria in a sample of 60-74–year-old Norwegians. The observed total energy intakes suggest that several of the participants underreported their intake during the completion of the dietary recall. Implementing web-based dietary assessment tools in older adults is feasible, but guidance and support might be needed to ensure valid completion.

## Introduction

A healthy diet throughout the life course improves health and reduces the risk of disease [1]. The World Health Organization’s Thirteenth General Programme of Work (GPW13) [2] emphasizes the encouragement of healthier life choices and behaviors including improvements in diet. The Norwegian National Action Plan for a Healthier Diet involves multiple objectives [3] in line with the United Nations (UN) Decade of Action on Nutrition 2016-2025 [4]. A key point is the need for research to enhance knowledge of the relation between diet and health [3].

Life expectancy is increasing, and the world’s population is ageing [5]. Therefore, middle-aged and older adults are important target groups for monitoring food and nutrient intake as well as for dietary interventions to ensure healthy ageing. Information on food and nutrient intake can be collected from a variety of sources, including objective methods such as direct observation or analysis of biomarkers, and self-report methods, such as food frequency questionnaires (FFQs), 24-hour recalls, or food records. All methods have methodological limitations [6-8], including high costs and participant burden, and none are essentially optimal to accurately capture all elements of diet. Objective measurements, such as biomarkers, are not influenced by the participant’s personal beliefs or perceptions. However, biomarkers do not cover all components of the diet and are affected by absorption and metabolism; hence, they are not always suitable or feasible and are dependent on the study context [7]. Self-report tools, such as FFQs assessing habitual intake, food records, or dietary recalls are noninvasive and usually feasible to use in a variety of research settings. However, self-reported measures are prone to large measurement errors occurring from the knowledge and beliefs of the study participants and rely on long- or short-term memory [7]. The 24-hour dietary recall is considered one of the most accurate self-report tools, as it collects information about diet within a short recent period; however, repeated measurements are required to capture the usual diet. New technology provides increased convenience for using the 24-hour dietary recall [6] and improves their usage in large-scale studies.

Measure Your Food On One Day (myfood24) is a self-administered web-based dietary data collection tool and analysis software developed [9] and validated [10] at the University of Leeds, the United Kingdom. The tool can be set up as a 24-hour dietary record or recall, for one or multiple days of prospective or retrospective recording. The myfood24 software can be used not only as a research instrument but also for teaching or clinical use. Region-adapted versions of myfood24 already exist for use in Australia, the Caribbean, Denmark, France, Germany, the Middle East, and Peru. The use of the same tool enables between-country comparisons. A Norwegian version of myfood24 [11] was developed and finalized in 2020 with further updates in 2021. The adaption from the original UK version consisted mainly of adapting the food database and images in the data bank to the Norwegian food cuisine, and the portion size images used in the Norwegian version has shown favorable properties in young adults [11].

The UK version of myfood24 has demonstrated reasonable response proportions for repeated 24-hour dietary recalls in 60-85–year-old individuals in the United Kingdom [12], and the German version has shown satisfactory usability in adults aged ≥65 years who were provided instructions and assistance during a study visit [13]. The usability of the Norwegian version has yet to be examined. There is an overall need for studies on the usability of web-based dietary data collection tools in the older adult population, as such data are scarce. It is of particular interest to examine the properties of the newly developed Norwegian version of myfood24, as this has not been examined before. Therefore, we aimed to examine the usability of a web-based 24-hour dietary recall tool, the Norwegian version of myfood24, in a sample of Norwegian older adult women and men who have been provided no prior tutoring or instructions.

## Methods

### Study Design and Participant Recruitment

We recruited participants by combining targeted convenience and snowball sampling to ensure an even distribution of sex, age (between 60-74 years), and educational level (elementary school, high school, or other, or higher or university education). Only individuals with an email address and access to a smartphone, tablet device, or computer were invited. We approached individuals accessible to the researchers through their personal networks (convenience sampling), and further invited individuals approached by already recruited individuals (snowball sampling). A total of 72 (convenience sample size) women and men aged 60-74 years (58% women) were invited to complete a single dietary recall. Invitations were sent in 3 rounds between May 12 and June 8, 2021, of which the first 2 groups received 1 reminder after 2 weeks (reminders were sent to all, as participation was anonymous).

### Instruments

#### myfood24 24-Hour Dietary Recall

myfood24 [9,10] dietary recall can be completed on the internet using a smartphone, tablet device, or computer. The participants were asked to report the intake of food during the previous day (24 hours), a so-called 24-hour dietary recall. The myfood24 is partially based on a multiple pass method, including an optional first quick list, a foods search in addition to prompts both for forgotten foods (for commonly forgotten foods and those often combined with other foods) and a final review before submission of the recall [9]. Participants can search for and add food items from a detailed list of foods primarily based on the Norwegian Food Composition Table 2019 [14] and chose portion sizes from pictures, natural measures (eg, number of potatoes), or household measures (eg, number of spoons of sugar added), as well as in an open-text response option. The portion sizes are a combination of pictures from the original UK version and the newly developed Norwegian version [11]. Based on the reported food intake, the total energy, macronutrient, micronutrient, and food intake were calculated in myfood24 (except for information from the open-text response).

#### System Usability Scale

The System Usability Scale (SUS) is a postsession questionnaire [15] and one of the most commonly used usability scales to evaluate a variety of products and services including hardware, software, mobile devices, and websites. The user (study participant in this case) is asked to evaluate the system (myfood24 in this case) after a user test (completing the myfood24 web-based dietary recall in this case). The SUS has 10 items answered with a 5-point Likert scale from “Strongly disagree” to “Strongly agree,” with alteration between positive and negative items to prevent response biases [16]. The 10 SUS items were as follows: (1) “I would like to use myfood24 again,” (2) “I found myfood24 was unnecessarily complicated,” (3) “I thought myfood24 was easy to use,” (4) “I needed the support of a technical person to use myfood24,” (5) “I found the various functions in myfood24 were well integrated,” (6) “I thought there was too much inconsistency in myfood24,” (7) “I imagine most people would be able to use myfood24 very quickly,” (8) “I found myfood24 very cumbersome (awkward) to use,” (9) “I felt very confident using myfood24,” and (10) “I needed to learn a lot of things before I could get going with myfood24.”

### Data Collection

The invitees were sent an email with a weblink to myfood24 and an SMS text message to their mobile phone with information that the email had been sent. The email included a brief description of the aim of the study (ie, to study the usability of a web-based dietary data collection tool) and a common (ie, not individual) weblink to complete the 24-hour dietary recall and a questionnaire about usability and demographics. No detailed guidance about how to complete the myfood24 was provided; that is, no preparation or tutoring.

By clicking the weblink in the email, the participant was directed to myfood24 and asked to report all food and drink intake from the previous day. After completion of the 24-hour dietary recall (ie, by clicking “submit” in myfood24), the participant was automatically redirected to a separate external web-based questionnaire created using Nettskjema [17], a web-based questionnaire tool resource developed by the University of Oslo, Norway. The Nettskjema questionnaire included the 10 SUS items, 3 related to demographic characteristics—sex, age group (60-64, 65-69, or 70-74 years), and education level (“What is your highest level of education?” with response alternatives of “elementary school/high school/other” or “higher education/university”)—along with 1 question about the device used to complete myfood24 (“smart phone,” “iPad/tablet,” or “PC/computer/Mac”) and an open-ended question (“Do you have other comments?”) where the participant could add supplemental information (as free text). Demographic data from Nettskjema could not be linked to the information about dietary intake from myfood24.

### Ethics Approval and Consent to Participate

According to the Norwegian Health Research Act [18], analysis of anonymous data does not require ethical approval; therefore, this study is not evaluated by the Regional Committees for Medical and Health Research. In this study, true anonymity was ascertained through the following procedures: (1) the weblink to the questionnaires (sent to the participants’ personal emails) was a nonpersonal URL (ie, all participants received the same weblink with no possibility to identify individuals), (2) the demographic data collected were aggregated (ie, group-level data for age and education), and (3) demographic variables from Nettskjema could not be linked to the information about dietary intake from myfood24. The email to all participants included information about the study. Completion of the questionnaire was regarded as consent.

### Data Analysis

The SUS score was calculated in accordance with Sauro 2011 [16]. The scoring is 0-4 for each item, where for odd items (positive) 1 is subtracted from the user response, and for even-numbered items (negative) 5 is subtracted from the user response, added up and multiplied by 2.5 (to convert the score to 0-100), with a score of 68 points or higher considered a “satisfactory product” [16]. We examined the mean (SD) SUS score and median (IQR) values as means as well as proportions within the satisfactory product cut-off limit in groups stratified by sex, age (60-69 and 70-74 years), and education. We used Stata (StataCorp LLC) to calculate study sample characteristics, including energy intakes, as well as total energy intake in kJ, including proportions with energy intakes outside standard cut-offs of <2100, >16,800 [19], and >8000 kJ (ie, 500, 4000, and 1900 kilocalories, respectively), mean and energy percentages (E%) for macronutrients, and mean SUS scores with 2-sample *t* test for differences between age groups (normal distribution observed with histograms). We used Excel (2016; Microsoft Corp) to present the response to each usability item as frequencies. Participants’ free-text comments were grouped into themes.

## Results

A total of 60 women and men participated (participation 83%, 57% women). Study sample characteristics and energy intakes are shown in Table 1. Half of the participants were in the oldest age group. Two-thirds reported having higher education, equally distributed by age. To complete myfood24, 41% (n=24) used a smartphone, 18% (n=11) used a tablet device, and 41% (n=24) used a computer. Two participants had not recorded any foods or beverages in myfood24. When excluding these, the mean and median total energy intake calculated from myfood24 were 5815 (SD 3093) kJ and 5896 (IQR 3815-7089) kJ, respectively, for the overall study sample. In total, 14% (n=8) had an energy intake of <2100 kJ, 17% (n=10) had an energy intake of >8000 kJ, and none had an energy intake of >16,800 kJ. The E% for all macronutrients were within the range of the national recommendations. In total, 10 participants recorded food items in the open-text option (eg, “home-made bun with spread for lunch” and “cloudberries with cream for dessert”).

**Figure 1:**
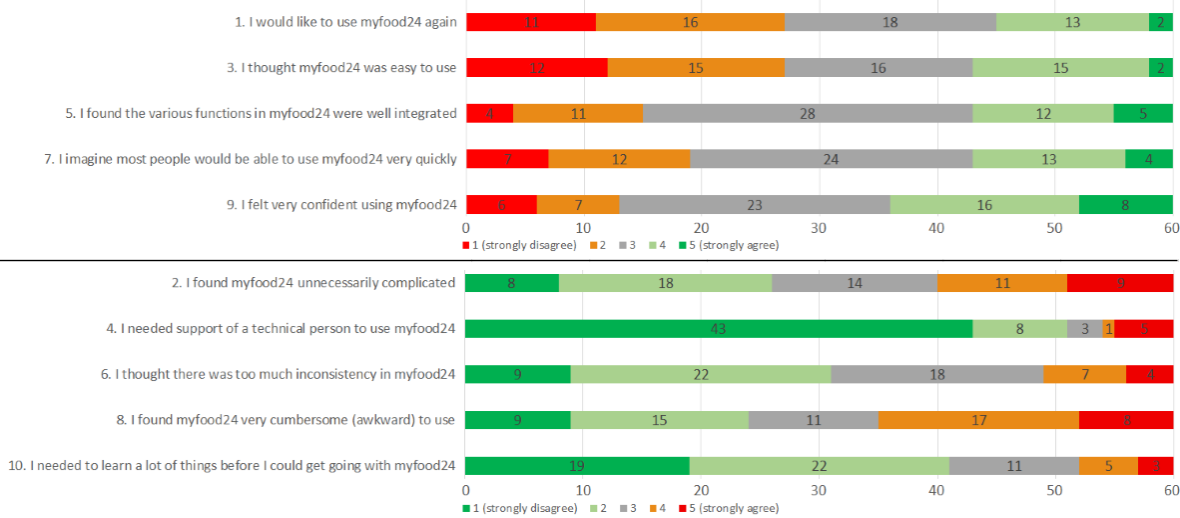
Distribution (frequency) of the response to each System Usability Scale item after completion of myfood24.

The response to each SUS item is presented in Figure 1, where the positive items (the 5 odd-numbered questions) and the negative items (the 5 even-numbered questions) have been grouped to enhance readability. The positive items were highly correlated, with responses 25%-28% above the middle category (agree or strongly agree), except for the item “I felt very confident using myfood24,” where 40% (n=24) responded above the middle category (agree or strongly agree). Two negative statements that stood out were “I needed support of a technical person to use myfood24®” where 72% (n=43) responded with “strongly disagree” and in total 85% (n=51) responded below the middle response category (disagree or strongly disagree), and “I needed to learn a lot of things before I could get going with myfood24®” where 68% (n=41) responded below the middle category (disagree or strongly disagree).

A total of 16 participants (27%) had SUS scores of ≥68 (ie, within the satisfactory product cut-off). Table 2 presents mean SUS scores in women and men, low and high educational groups, and smartphone, tablet device, and computer users by age group. The mean and median overall SUS scores were 55.5 (SD 18.6) and 55.0 (IQR 41.3-70.0), respectively, with higher scores in the younger age group (*P*=.02). For each group stratified by sex, education, and device used, there was a tendency toward higher SUS scores in the younger compared to the older age group, but only significantly different among those with higher education.

**Table 1:** Participant characteristics and energy intakes for the overall sample (N=60) and by age group.

Characteristics		Total	Age group 60-69 years (n=30)	Age group 70-74 years (n=30)
Women, n (%)		34 (56.7)	19 (63.3)	15 (50.0)
Higher education, n (%)		44 (73.3)	21 (70.0)	23 (76.7)
**Device, n (%)**				
	Smartphone	24 (40.7)	13 (43.3)	11 (37.9)
	Tablet device	11 (18.6)	4 (13.3)	7 (24.1)
	Computer	24 (40.7)	13 (43.3)	11 (37.9)
Total energy intake^a^ (kJ), mean (SD)		5815 (3093)	—^b^	—
Carbohydrates (energy percentage), mean (SD)		41.7 (11.1)	—	—
Protein (energy percentage), mean (SD)		19.9 (6.0)	—	—
Fat (energy percentage), mean (SD)		32.9 (11.6)	—	—
Alcohol (energy percentage), mean (SD)		2.7 (5.9)	—	—
Fiber (energy percentage), mean (SD)		2.7 (1.3)	—	—

^a^Energy and nutrient intakes were only calculated for participants with a total energy intake of >0 kJ (n=58). One participant did not report the device used.

^b^—: not available.

**Table 2:** Mean usability score^a^ for the overall sample (N=60) and by age group.

Categories	Total, mean (SD)	Age group 60-69 years (n=30), mean (SD)	Age group 70-74 years (n=30), mean (SD)	*P* value^b^
Total	55.5 (18.6)	61.3 (17.6)	49.8 (18.0)	.02
Women	56.3 (20.5)	61.6 (18.4)	49.5 (21.7)	.09
Men	54.5 (16.0)	60.7 (17.0)	50.0 (14.1)	.09
Lower education	62.0 (14.4)	65.6 (15.0)	57.5 (13.2)	.28
Higher education	53.1 (19.5)	59.4 (18.6)	47.4 (18.9)	.04
Smartphone	52.5 (20.1)	61.9 (14.8)	55.5 (10.8)	.24
Tablet	55.9 (25.1)	68.1 (28.1)	48.9 (22.3)	.24
Computer	59.0 (13.3)	58.5 (17.6)	45.5 (21.5)	.12

^a^A System Usability Scale score of ≥68 indicates that the usability criteria were satisfied.

^b^A 2-sample *t* test was performed to assess differences between age groups.

In total, 18 participants had left comments in the free-text option. These comments (examples in parentheses) could thematically be grouped to (1) lack of categories of meals or foods (“Found nowhere to register supper”), (2) complexity (“Unnecessary complex at the same time as not nuanced enough to catch important differences between foods”), (3) wording (“Why is the word ‘generic’ used?”), or (4) technical problems (“Two times nearly finished, got kicked out”).

## Discussion

### Principal Findings

In this study of the usability of a new web-based dietary assessment tool among older adult Norwegian women and men who were provided no guidance or support, we found the overall usability to be less than satisfactory and the overall total energy intake to imply error (underreporting) in completion of the dietary recall. In total, 17% of participants had energy intakes above 8000 kJ, and 27% had usability scores within the range indicating a satisfactory product.

The E% for all macronutrients were within the recommendations from the Norwegian Health Directorate [20], but in the total sample, the calculated mean total energy intake was only 60% of the daily total energy intake of an average inactive adult. This could imply that, for most participants, a variety of foods were recorded, but some foods (or meals) were probably omitted from the recording, or the amounts reported were incorrect. A traditional 4-meal Norwegian diet consists of breakfast (typically bread with spread or cereal with milk or yogurt), lunch (typically sandwiches), dinner (typically a dish with vegetables and meat or fish), and a light supper (typically similar foods as in breakfast or lunch), while myfood24 is based on 3 main meals, and other meals are classified as snacks. Based on the qualitative feedback provided in the open-text fields, some of the participants seem to struggle with this difference. Further, a total of 16% recorded food in the open-text response (instead of finding an alternative in the foods list), and these foods were omitted from the calculation of energy intake. Including these items in the calculations would have increased the mean energy intake. Misreporting is, however, common in all self-administered tools for all age groups, and for dietary assessment tools most often in the direction of underreporting, leading to incorrectly low estimates of total energy intakes.

Our results deviate considerably from the findings from a usability study of the German version of myfood24 [13], who found a median SUS score above the good product cut-off (median 74, IQR 70-83) among older adults aged ≥65 years, as well as total energy intake within the normal range, implying that the study participants completed myfood24 correctly. A potential explanation for this difference could be that the data collection in the German study was performed during a study center visit, where the participants received instructions on how to complete myfood24 as well as on-site assistance [13]. Further, the participants completed 3-4 consecutive recalls (ie, probably resulting in the participant being acquainted with the tool), 11% had previous experience in completing 24-hour dietary recalls, and 21% had a nutritional or food science background [13], of which both are expected to increase the perception of usability. Furthermore, the German version of myfood24 contains a higher number of foods than the Norwegian version, including a large number of branded foods. This can make it easier to determine the exact food consumed, thus increasing usability. However, there is a need for balance in the number of foods listed. If the list of foods is regarded as too extensive by the user, this can decrease usability.

During the development of the original UK version of myfood24, a small sample of adults aged above 65 years was included to report expectations from myfood24 [10]. The sample’s median SUS score was poor (29, IQR 63); however, this sample consisted of only 4 participants [9]. Thus, the findings could have resulted from chance events. A review of another web-based 24-hour recall tool, the Automated Self-Administered 24-hour (ASA24) Dietary Assessment Tool used in Canadian populations found that older adults encountered technical challenges and emphasized the need for tailored support [21]. However, the need for technical assistance was not limited to older adults, and although older adults had a lower technical troubleshooting capacity, they simultaneously were willing to spend more time troubleshooting than younger adults [21]. There is strong reason to believe that with increasing ownership of smartphones and tablets as well as preference to use digital technologies in everyday life, the feasibility of using web-based dietary assessment tools will also increase in the older adult population [6].

Nonetheless, digital illiteracy is a concern for self-administered web-based assessment tools, particularly among older adults. In a feasibility study of myfood24 among 60-85–year-old individuals in the United Kingdom [12], those completing multiple recalls reported higher self-confidence with technology and had a higher technology readiness score than those who did not complete any recalls. Notably, the participants in the UK study were recruited from an ongoing cohort study of cognitively healthy older adults [12], which could introduce a risk of selection bias.

Even though the overall SUS score in our study was lower than that considered satisfactory, we do believe that myfood24 is a useful tool to measure dietary intake. The participants in our study were solely provided information about the aim of the study, with no guidance or support in completing the recall. We believe that additional tailored instruction on how to complete the recall could have improved the overall perception of usability considerably and led to less underreporting. Moreover, usability questions highlighting the need for technical support or training revealed that technical challenges did not constitute a major barrier. Thus, we believe that technical difficulty was not the main contributor to the observed SUS score. In telephone surveys by Statistics Norway, 70% of 75-79–year-old individuals reported daily use of the internet in 2021 compared to 40% in 2015 [22], implying that digital illiteracy is decreasing in Norwegian older adults. Therefore, we believe that the feasibility of using web-based assessment tools in older adults will increase substantially over the next few years. More work is needed in adapting the Norwegian version of myfood24 to older adults, such as explaining how to record supper. Based on the qualitative feedback from comments provided by the participants in this study, we also believe that adding more foods and more synonyms to the foods list would improve the user experience. Previous usability studies of self-administered dietary assessment apps among older adults, include apps with voice-added reporting [23] or apps where health personnel can complete the reporting on behalf of the respondent [24]. For older adults with impairments, such modifications are helpful. Other apps are developed for clinical use by health personnel only. In addition to being a self-administrative tool for research or teaching purposes, myfood24 provides a separate health care solution for health personnel to set patients’ personalized nutrient targets and track their progress. Thus, modification to fit the context and the need of the individual is possible when using myfood24.

### Strengths and Limitations

The study was set up to be anonymous to protect participant privacy; therefore, the participants received unidentifiable weblinks. Thus, a major study limitation is that we could not link SUS scores and dietary intakes on an individual level, to examine the correlation between intake and SUS scores. Further, we collected no information regarding whether the participants were comfortable with using technology. We invited only women and men with an email address and access to a device to complete the recall, and we did not examine reasons for nonparticipation. People without email addresses or those who did not participate could be less digitally literate. Thus, lower SUS scores could be anticipated among nonparticipants. A strength of the study is testing of the tool in a mixed group of older adults (ie, both sexes, different ages, and educational levels).

### Conclusions

In this population of older adult Norwegian women and men, the overall usability score for a web-based 24-hour dietary recall assessment tool, myfood24, was lower than what is considered satisfactory according to standard usability criteria. Implementing myfood24 in older adults is feasible, but additional guidance and support might be needed to ensure valid completion, and more work is needed for adapting myfood24 for use among older adult Norwegians.

## Abbreviations

ASA24: Automated Self-Administered 24-hour
E%: energy percentage
FFQ: food frequency questionnaire
GPW13: World Health Organization’s Thirteenth General Programme of Work
myfood24: Measure Your Food On One Day
SUS: System Usability Scale
UN: United Nations
